# TOPK is highly expressed in circulating tumor cells, enabling metastasis of prostate cancer

**DOI:** 10.18632/oncotarget.3630

**Published:** 2015-03-20

**Authors:** Huimin Sun, Lei Zhang, Changhong Shi, Peizhen Hu, Wei Yan, Zhe Wang, Qiuhong Duan, Fan Lu, Lipeng Qin, Tao Lu, Juanjuan Xiao, Yingmei Wang, Feng Zhu, Chen Shao

**Affiliations:** ^1^ Department of Urology, Xijing Hospital, The Fourth Military Medical University, Xian, China; ^2^ Department of Epidemiology, Faculty of Preventive Medicine, The Fourth Military Medical University, Xian, China; ^3^ Laboratory Animal Center, The Fourth Military Medical University, Xian, Shaanxi, China; ^4^ Department of Pathology, Xijing Hospital, The Fourth Military Medical University, Xian, China; ^5^ Department of Biochemistry and Molecular Biology, School of Basic Medicine, Huazhong University of Science and Technology, Wuhan, China; ^6^ Department of Biochemistry, Department of Basic Medicine, The Fourth Military Medical University, Xian, China; ^7^ PLA Lhasa General Hospital, Lhasa, China

**Keywords:** circulating tumor cells, T-LAK cell-originated protein kinase (TOPK), prostate cancer, metastasis

## Abstract

Circulating tumor cells (CTCs) are important for metastasis in prostate cancer. T-LAK cell-originated protein kinase (TOPK) is highly expressed in cancer cells. Herein, we established a xenograft animal model, isolated and cultured the CTCs, and found CTCs have significantly greater migratory capacity than parental cells. TOPK is more highly expressed in the CTCs than in parental cells and is also highly expressed in the metastatic nodules caused by CTCs in mice. Knocking down TOPK decreased the migration of CTCs both *in vitro* and *in vivo*. TOPK was modulated by the PI3K/PTEN and ERK pathways during the metastasis of prostate cancer. High levels of TOPK in the tumors of patients were correlated with advanced stages of prostate cancer, especially for high-risk patients of Gleason score≥8, PSA>20ng/ml. In summary, TOPK was speculated to be one of a potential marker and therapeutic target in advanced prostate cancer.

## INTRODUCTION

Metastasis is a multi-step process leading to the spread of neoplastic cells to distant sites. The mortality of prostate cancer patients is caused by metastatic deposit in many other tissues and organs, especially in bones and lymph nodes [1]. During metastasis, circulating tumor cells (CTCs) are generated from the primary tumor, and they subsequently invade and colonize distant organs [2]. The presence of CTCs in the blood of patients is a major indicator of metastasis. The CTC population is highly resilient, enabling the cells to colonize a foreign microenvironment [3, 4]. Therefore, it is important to monitor the number of CTCs in the blood of a patient.

In the early stages of the cancer, CTC numbers can be used to predict the risk of tumor metastasis. During therapy, CTC numbers may be useful in evaluating therapy response or in choosing a suitable therapeutic regimen [5-9]. The number of CTCs prior to therapy initiation, during, and after therapy, is indicative of the length of progression-free survival (PFS) and of overall survival (OS) [10-13]. Furthermore, new biological therapeutics that selectively and specifically target CTCs may be developed through the molecular analysis of CTCs [7, 14]. Clinically, CTCs have been identified as an independent prognostic marker in a number of metastatic cancers [15-16], and a growing amount of evidence suggests that CTC monitoring can identify those patients who are responding to or failing therapy early in the course of a treatment [17].

CTCs have been used to detect prostate cancer metastasis and predict the overall survival in patients with metastatic prostate cancer [18, 19], thus, further characterizing CTCs may clarify the role of tumor shedding in patients with intermediate- and high-risk prostate cancer [20, 21].

T-LAK cell-originated protein kinase (TOPK) belongs to the dual specific serine/threonine kinase MAPKK family, between MEK1/2 and MEK7 [22]. It is highly expressed in some cancers, such as colorectal cancer, breast cancer, and melanoma, and in some fetal tissues, but is lowly or not expressed in normal tissues.

Many studies have demonstrated that TOPK plays an important role in regulating diverse cellular processes, including the proliferation of neural progenitor cells and the development and progression of a variety of human tumors [23-30]. Elevated levels of TOPK may be associated with poor prognosis in a variety of malignant cancers, including hematological cancers, melanoma, colorectal cancers, neuroblastic tumors, and renal cell carcinoma [23-27]. TOPK has also been recognized as a metastasis-promoting kinase in lung cancer metastasis [28].

Extracellular signal-regulated kinases (ERK) have been identified as one of the protein substrates of TOPK/PBK. Zhu *et al.* demonstrated that positive feedback between TOPK and ERK2 promotes the tumorigenic properties of colorectal cancer cells [30]. Moreover, Shih *et al.* showed that TOPK promotes cell migration by modulating the PI3K/PTEN/AKT pathway in lung cancer [28].

CTCs are difficult to detect because of their scarcity and biological heterogeneity. Although TOPK is highly expressed in numerous kinds of tumors, its role in the metastasis of prostate cancer has not been elucidated. In this study, CTCs from prostate cancer were isolated and cultured, and the role of TOPK in the migration of prostate cancer CTCs was studied. Given that TOPK is significantly upregulated in CTCs of prostate cancers and promotes CTC migration and/or invasion, these findings suggest TOPK as a target for therapy and a prognostic marker for metastatic prostate cancer.

## RESULTS

### CTCs are more malignant than PC3 cells

CTCs are a highly heterogeneous population of cancer cells that detach from primary tumors and enter the bloodstream, enabling them to colonize a foreign microenvironment, resulting in tumor metastasis.

In this study, CTCs were isolated and cultured according to the methods that have been reported [31, 32], and RBCs were lysed during the isolation. The isolated CTCs were cultured and frozen for future use.

**Figure 1:**
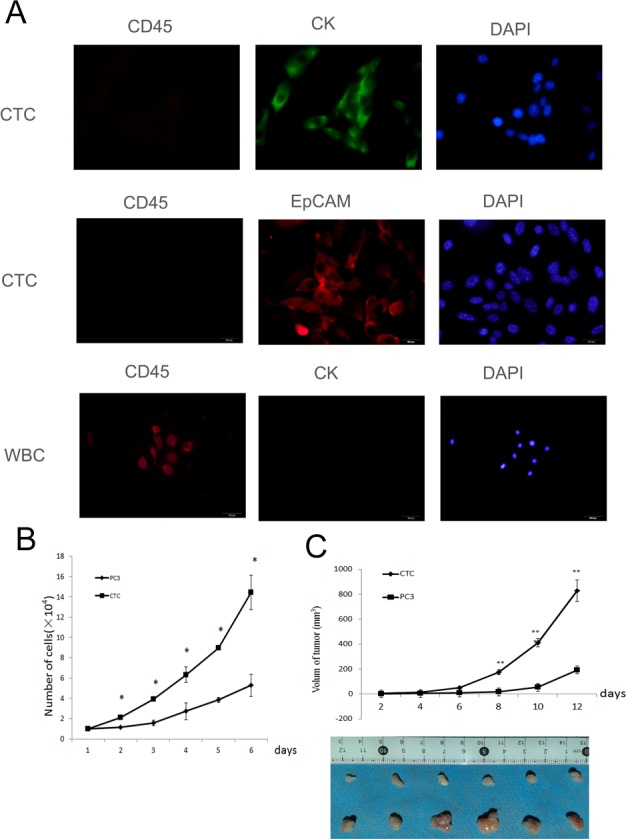
Isolated and cultured CTCs proliferate faster than PC3 cells **A**. The isolated cells were identified as CTCs by immunofluorescent staining for EpCaM, CK 19, and CD45. **B**. The proliferation of CTCs and PC3 cells *in vitro*. The CTCs grew faster than PC3 cells. **C**. The proliferation of CTCs and PC3 cells *in vivo*. The proliferation of CTCs was faster than PC3 cells *in vivo*. Data represent the mean ± SD from three independent experiments, *p < 0.05, **p < 0.01.

To demonstrate that the isolated cells were CTCs, the cells were stained for the transmembrane protein EpCAM and cytoplasmic keratin 19 (CK19), which are ubiquitously expressed in CTCs [33-35]. The cells were also stained for common leukocyte antigen (CD45) to exclude possible leukocyte contamination [36, 37]. Immunofluorescence showed that the isolated cells were positive for EpCAM and CK19 and negative for CD45 (Figure 1A), indicating that the cells were CTCs.

Next, the proliferation of CTCs and PC3 cells was tested. Comparison of the growth curves of CTCs and PC3 cells demonstrated that CTCs grew faster than PC3 cells (Figure 1B). Furthermore, the proliferation of CTCs or PC3 cells were compared *in vivo* after subcutaneously injecting 3×10^6^ cells into the flanks of mice. The tumors of mice injected with CTCs grew from 0 mm^3^ to nearly 1000 mm^3^ within two weeks, whereas tumors grew much slower in mice injected with PC3 cells (Figure 1C). In summary, the above results demonstrate that CTCs proliferate faster both *in vitro* and *in vivo* when compared to their parental PC3 cells.

### The metastatic capacity of CTCs is stronger than parental PC3 cells

**Figure 2:**
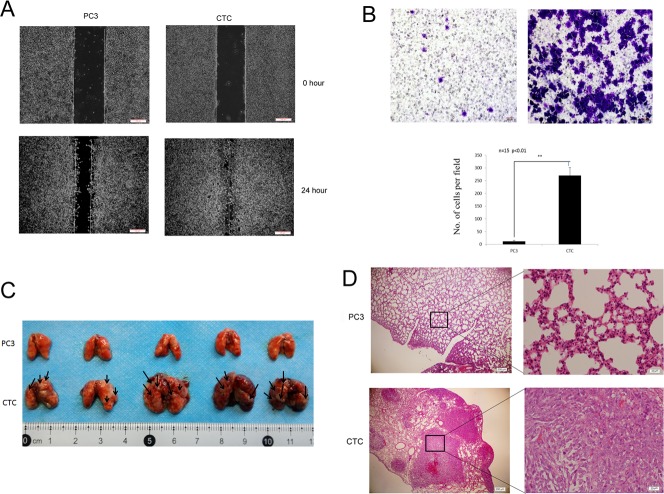
CTCs have greater metastatic capacity than parental PC3 cells **A**. Scratch wound assay demonstrating that CTCs migrate faster than PC3 cells. The dotted lines show the area where the scratch wound was created. The scratch wound assay was performed in quadruplicate. **B**. Transwell invasion assay. CTCs have greater invasive capacity than PC3 cells. Representative images from Transwell invasion assays of PC3 cells (left) and CTCs (right) cells are shown. The results are presented as mean ± SD of 3 independent experiments (*P* < 0.01). **C**. Lung metastasis in mice was assessed 14 days following the administration of either CTCs or PC3 cells via intravenous injection. Grossly visible metastatic nodules were abundant in the CTC group (bottom), but were not obvious in the PC3 group (top). (D) Hematoxylin/eosin-stained lung sections from mice injected with either CTCs or PC3 cells. Poorly differentiated adenocarcinoma cell clusters were found in the lungs of mice in the CTC group. Magnification, 40× and 400×.

The presence of CTCs in blood vessels is a major step in metastasis, and CTCs are closely related to metastasis [1, 2]. Therefore, the migratory and invasive capacities of CTCs and PC3 cells were investigated. In rescue wound healing assays, the scratch created in a CTC culture was nearly completely healed within 24 hours, whereas only half of the scratch created in a PC3 cell culture healed (Figure 2A), suggesting that CTCs migrated faster than PC3 cells. Moreover, in Transwell assays, more CTCs invaded across the membrane than PC3 cells, demonstrating enhanced invasive capacity (Figure 2B).

Next, the migratory capacities of CTCs and PC3 cells were tested *in vivo*. Mice were injected intravenously via tail vein with 2×10^5^ of either CTCs or PC3 cells. Two weeks later, the entire lungs of the mice that were injected with CTCs contained metastatic tumors, whereas few tumors were found in the lungs of the mice that were injected with PC3 cells (Figure 2C). These data indicate that CTCs are more metastatic than PC3 cells both *in vitro* and *in vivo*.

### TOPK is highly expressed in CTCs

Together, the data in Figures 1 and 2 demonstrate that CTCs have remarkable proliferative and metastatic capacities. Numerous studies have shown that TOPK plays a crucial role in promoting the tumorigenesis [38] and proliferation of cancer cells [39]. Shih *et al*. reported that TOPK promotes lung cancer metastasis [29], and Wei *et al.* reported that TOPK is a potential prognostic predictor of stage I lung adenocarcinoma [40]; however, the role of TOPK in metastatic prostate cancer has not been investigated to date.

To test our hypothesis that TOPK might play an important role in the ability of CTCs to mediate prostate cancer metastasis, the expression of TOPK in CTCs was tested.

**Figure 3:**
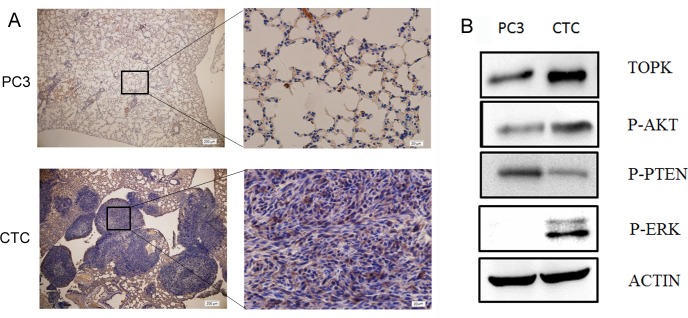
TOPK is highly expressed in CTCs **A**. Immunohistochemical analysis of TOPK expression in lung sections from mice injected with either CTCs or PC3 cells. Nuclear and cytoplasmic expression of TOPK was detected in the pulmonary metastatic CTCs, while no or very weak staining of TOPK was detected in normal pulmonary alveolar epithelial cells from mice in the PC3 group. **B**. Western blotting was performed to examine the expression of TOPK, phospho-PTEN, phospho-AKT, and phospho-ERK in PC3 cells and CTCs.

Immunohistochemical analysis of the lungs of mice intravenously injected with either CTCs or PC3 cells showed that TOPK was more highly expressed in the lungs of the mice that were injected with CTCs than in those injected with PC3 cells (Figure 3A). Moreover, Western blotting showed that TOPK was more highly expressed in CTCs than in PC3 cells (Figure 3B). Because TOPK has been shown to stimulate AKT-dependent cell migration/invasion by relieving PTEN-dependent suppressive effects [28], we also measured the expression of phospho-AKT and phospho-PTEN in the CTCs. These experiments showed that the CTCs had increased Ser473 phosphorylation of AKT and decreased Ser380/Thr382 phosphorylation of PTEN. Because ERK2 is the substrate of TOPK, and phospho-ERK2 is associated with metastasis [31], we next measured the level of phospho-ERK2 in CTCs. Increased ERK2 phosphorylation was observed. All these findings indicate that TOPK signaling is important in CTCs.

### Knocking down TOPK prevents CTC metastasis

**Figure 4:**
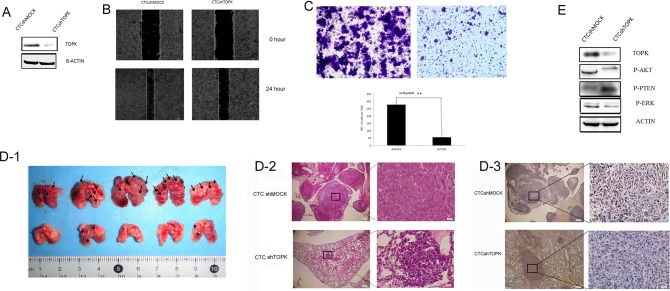
Knocking down TOPK in CTCs decreased their metastatic capacity **A**. Expression of TOPK in the CTCshTOPK and CTCshMOCK cell lines. **B**. Scratch assays performed in CTCshTOPK and CTCshMOCK cells showed that CTCshMOCK cells migrate faster than CTCshTOPK cells. **C**. Transwell assays of CTCshTOPK (right) and CTCshMOCK (left) cells showed that CTCshMOCK cells have greater invasive capacity than CTCshTOPK cells. **D**. The metastatic capacity of CTCshTOPK and CTCshMOCK cells *in vivo*. (D1) Gross examination revealed increased numbers of visible metastatic tumor nodules in the lungs of mice from the CTCshMOCK group compared to the lungs of mice from the CTCshTOPK group. Lungs of mice from the CTCshMOCK group also showed focal hemorrhages (dark red areas of lung surfaces). Arrows indicate tumor metastatic nodules. (D2) Hematoxylin/eosin-stained lung sections from mice injected intravenously with either CTCshMOCK or CTCshTOPK cells. In the CTCshMOCK group, metastatic tumors presented as multifocal nodules, whereas only focal microscopically visible metastases were found in the CTCshTOPK group. Magnification, 40× and 400×. (D3) Immunohistochemical analysis of TOPK expression in pulmonary metastatic nodules. CTCshTOPK metastatic tumor cells showed strong nuclear and cytoplasmic expression of TOPK, whereas normal pulmonary alveolar epithelial cells showed no or very weak expression of TOPK. Magnification, 40× and 400×. **E**. The expression of phospho-PTEN, phospho-AKT, and phospho-ERK in CTCshTOPK and CTCshMOCK cells.

In order to further confirm the role of TOPK prostate cancer metastasis, TOPK was knocked down in CTCs through lentiviral transfection. Western blotting demonstrated that the expression of TOPK in CTCshTOPK cells decreased much more remarkably than that in CTCshMOCK cells (Figure 4A).

Next, we examined the migratory and invasive capacity of CTCshMOCK and CTCshTOPK cells in rescue wound healing assays and Transwell assays. The results of rescue wound healing assays demonstrated the scratch created in a CTCshMOCK cell culture healed much faster than the scratch created in a CTCshTOPK cell culture (Figure 4B). Moreover, the results of Transwell assays indicated that more CTCshMOCK cells than CTCshTOPK cells invaded across the membrane within the same time period (Figure 4C). Together, these *in vitro* assays demonstrate that knocking down TOPK expression in CTCs results in dramatically decreased migratory and invasive capacity.

To test the role of TOPK in the metastatic potential of CTCs *in vivo*, mice were intravenously injected with 2×10^5^ of either CTCshMOCK cells or CTCshTOPK cells. Two weeks later, the pulmonary metastatic tumors were observed. Immunohistochemical staining showed the expression of TOPK was lower in the lungs of the mice that were injected with CTCshTOPK cells than in those injected with CTCshMOCK cells. Importantly, the CTCshMOCK group showed focal hemorrhages, and there were more visible metastatic tumor nodules in the CTCshMOCK group than in the CTCshTOPK group. These findings indicate that knocking down TOPK greatly decreased the ability of CTCs to form tumor metastases in the lung following intravenous injection (Figure 4D and 4E). Together, these assays reveal that TOPK plays a very important role in the metastasis of prostate cancer.

The expression of phospho-AKT, phospho-PTEN and phospho-ERK2 in the CTCshMOCK and CTCshTOPK cells were also detected. CTCshTOPK cells showed decreased Ser473 phosphorylation of AKT, increased Ser380/Thr382 phosphorylation of PTEN, and decreased phosphorylation of ERK2 when compared to CTCshMOCK cells (Figure 4F), further supporting the finding that knocking down TOPK inhibited metastasis in CTCs.

### TOPK expression is closely associated with prostate cancer grade

TOPK expression was examined by immunohistochemistry in 71 human prostate cancer and 30 benign prostatic hyperplasia (BPH) specimens with known clinical follow-up records. High expression of TOPK (scores of 2 and 3) was strongly associated with advanced prostate cancer (Table 1). According to The Guideline for European Association of Urological (EAU), Gleason score ≥ 8, PSA> 20 ng/ml or stage > T2c are the borderline range for high risk of prostate cancer metastasis [41]. Overexpression of TOPK in prostate cancer is associated with Gleason score ≥ 8 (P = 0.0016), PSA > 20 ng/ml (P = 0.02), and stage > T2c (p = 0.035).

**Table 1:** Relationship between TOPK expression and clinical diagnostic criteria in 71 prostate cancer patients

Variables	TOPK expression		P-value^a^	Fisher
	Low (0,1) (n=49)	High(2,3) (n=22)		
**Level of PSA(ng/ul)**			0.02	0.017
PSA≥20	15	13		
PSA<20	34	9		
**Gleason grade**			0.0016	0.0014
≤7	31	5		
≥8	18	17		
**TNM stage**			0.035	0.0233
Stage≥T2c	18	14		
Stage<T2c	31	8		

a P-values were derived with a two-tailed Pearson's x^2^ test and Fisher exact test.

## DISCUSSION

TOPK plays a role in the development and proliferation of many types of cancers, such as breast, lung, and colon cancers and melanoma, and it is associated with poor prognosis in multiple types of cancer [24-25, 28, 42-44]; however, no studies of the expression of TOPK in prostate cancer have yet been reported. Our study in xenograft animal model demonstrates that TOPK expression is closely associated with the development and proliferation of prostate cancer.

Shih's study demonstrated that TOPK promotes cell migration and invasion in lung cancer [28]. The combination of an integrated microarray with empirical analyses in Shih's study further suggests that TOPK could be a potential prognostic marker for lung cancer. Minoo's study of 399 CRCs showed that TOPK could be a marker of tumor in colon cancer in combination with CDX2, CD44v6, CD44s, nuclear ß-catenin, pERK, APAF-1, E-cadherin, p21, and bcl2 [45]. All of these results indicate that TOPK could be a potential tumor marker as well as an important therapeutic target.

**Figure 5:**
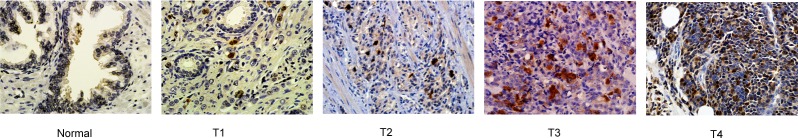
Elevated expression of TOPK is correlated with pathological grade in prostate cancer patients Normal prostate tissues showed no or very weak expression of TOPK, whereas prostate tumor tissues of high pathological grades showed strong expression of TOPK.

The results of our *in vitro* and in xenograft animal model experiments demonstrate that prostate cancer cells with high TOPK expression have increased migratory and invasive capacity (Figures 2 and 4), and knocking down TOPK decreased the metastatic potential of these cells. Furthermore, we detected differences in the expression of TOPK in BPH prostate tissue and prostate tumor tissues (Figure 5). TOPK expression was lowest in the BPH prostate tissues or tissues adjacent to prostate tumors and was significantly elevated in tumor tissues with high pathological grades (Figure 5). Our analysis of the relationship between TOPK expression and the major clinical diagnostic criteria used in advanced prostate cancer (PSA ≥ 20ng/ml, Gleason score ≥ 8, or Stage ≥ T2c) in 71 prostate cancer patients also indicated that elevated TOPK expression can be used as an independent prognostic factor in high-risk prostate cancer. However, more cases are required to further validate the role of TOPK before any conclusions can be drawn.

The study by Shih *et al.* investigated the signaling pathway by which TOPK promotes lung cancer metastasis [28]. Their study indicated that TOPK decreased the protein stability of PTEN by stimulating it to undergo proteasome-dependent degradation. However, it is possible that TOPK could promote cell migration through a PTEN/AKT-independent mechanism. Our study showed that when TOPK was highly expressed, phospho-AKT was also highly expressed, while phospho-PTEN was expressed at low levels (Figure 3B). When TOPK was knocked down, the expression of phospho-AKT was also downregulated, while that of phospho-PTEN was upregulated (Figure 4E). Zhu's study identified a positive feedback loop between TOPK and ERK2 [30]. Accumulating evidence indicates that the ERK pathway can promote the migration and invasion of cancer cells and is closely associated with metastasis [46-49]. Our study demonstrates that when TOPK is highly expressed in CTCs, phospho-ERK is also highly expressed (Figure 3B), and when TOPK is knocked down, the expression of phospho-ERK was also downregulated (Figure 4E). These results are consistent with the previous studies and further indicate that TOPK signaling pathway can promote prostate cancer metastasis by regulating the PTEN/AKT signaling pathway and the ERK pathway. Importantly, all of these pathways have been shown to regulate the growth and metastasis of cancer.

Molecular targeted therapy has been applied to chronic myeloid leukemia, non-small cell lung cancer, colorectal cancer, gastrointestinal stromal tumors, breast cancer, and other tumors [50-58]. Currently, molecular targeted therapies for prostate cancer undergoing clinical trials include therapies against prostate-specific membrane antigen (PSMA) [59] and prostate stem cell antigen (PSCA) [60], anti-angiogenesis drugs, and anti-tumor cell signaling COX-2 inhibitor drugs [61]. However, at this time, there is no evidence to support the use of molecular targeted therapy in prostate cancer.

Recently, targeted inhibitors against TOPK have been reported, such as HI-032 [62] and OTS964 [63]. In our study in cells and in xenograft animal model, when TOPK was highly expressed, both the cancer cells and the tumors grew faster (Figure 2), the proliferation and development of the cancer cells and tumors were inhibited when TOPK was knocked down (Figure 4). This data suggest that TOPK might be a useful therapeutic target in prostate cancer, inhibiting TOPK might be a useful therapeutic approach in primary and metastatic prostate cancer. Therefore, it is crucial to develop new TOPK inhibitors for prostate cancer targeted therapy.

Numerous reports have demonstrated that the presence of CTCs in the peripheral blood of patients with various forms of metastatic carcinomas is positively associated with poor clinical prognosis [9]. Considering that biopsy is an invasive and painful procedure, the evaluation of CTCs in the blood might represent a potential alternative diagnostic method. Thus, CTCs may become a novel tool for optimizing diagnosis and therapy for patients with early and metastatic cancers [64-66].

Although many methods have been reported for detecting CTCs in the peripheral blood of cancer patients [65-70], there are only a few approaches in use to isolate and culture CTCs [31, 32]. In this study, we improved and simplified the RBC lysis method for isolating and culturing CTCs. The cells that are isolated with this method can be cultured and passaged continually, making it possible to study the characteristics of CTCs using cultured and passaged CTCs. The approach is quite stable and repeatable and provides easy access to CTCs for researchers.

Based on our findings, high expression of TOPK in CTCs and the experiment in xenograft animal model indicated that TOPK is associated with the metastasis of prostate cancer. We propose that TOPK may serve as a diagnostic marker of high-risk prostate cancer. Moreover, TOPK could be a potential therapeutic target in prostate cancer.

## MATERIALS AND METHODS

### Cell culture

The PC3 prostate cancer cell line was purchased from American Type Culture Collection (ATCC, Rockville, MD, USA). The cells were maintained at 37°C in RPMI 1640 medium supplemented with 10% fetal bovine serum (FBS) in a 5% CO_2_ incubator, following the instructions provided by ATCC.

### Xenograft generation

Non-obese Diabetic/Severe Combined Immunodeficiency (NOD–SCID) mice were purchased from Beijing HFK Bioscience CO., LTD (Beijing, China). The animals were housed in micro-isolator cages with autoclaved bedding and provided autoclaved food and water. A total of 20 NOD/SCID male mice received injections of 8×10^5^ PC3 prostate cancer cells in 80 μl phosphate-buffered saline (PBS; pH 7.4) into their prostates. The control mice were injected with PBS into the same location. The mice were euthanized for isolation of CTCs eight weeks later.

Athymic Balb/c nude mice were purchased from BEIJING HFK BIOSCIENCE CO., LTD (Beijing, China). PC3 cells or CTCs (3×10^6^ cells suspended in 200 μl PBS) were injected subcutaneously into the right flank of athymic Balb/c nude mice in order to assess the tumor formation capacity of CTCs. The length (l), width (w), and height (h) of the tumor were measured every other day. The volume (V) of the tumor was calculated using the following formula: V = 0.52 (l×w×h). The tumors were dissected and sent for immunohistochemical analysis at the Department of Pathology in Xijing Hospital. All animal experiments were performed following protocols approved by the Laboratory Animal Center of the Fourth Military Medical University.

### Orthotopic prostate transplantation

Mice were anesthetized with 40mg/kg pentobarbital by intraperitoneal injection. Under sterile conditions, a 0.6~0.8 cm incision was made along the ventral midline abdominal wall. The bladder and seminal vesicles of both sides were gently raised with tweezers, exposing the prostate. The cells were injected at the front and rear sides of the left and right lobes of the prostate, total 4 points, 2×10^5^cells in 20μl PBS (PH = 7.4) were injected into every point. After ensuring nothing was spilled, the abdominal muscles were sutured, the incision was closed, the skin wound was disinfected, and the mice were returned to their cages after waking.

### Isolation of circulating tumor cells

As soon as the mice were euthanized, blood was drawn by means of cardiac puncture following published protocols for obtaining CTCs from mouse blood [31, 32]. The mouse blood (200–900 μl per mouse) was pooled into a 2-ml EDTA anticoagulant tube on ice immediately after it was drawn. A 2-ml sample of packed blood cells was lysed with 13 ml of 1 × RBC lysis buffer (165 mM NH_4_Cl, 10 mM KHCO_3_, 0.1 mM EDTA) at room temperature within 10 minutes in a conical tube and then centrifuged for 8 minutes at 1,400 rpm at room temperature in a Central CL2 centrifuge (Thermo IEC). The cells were washed twice by resuspending the pellets in 6 ml of 1×PBS and centrifuged at 1,400 rpm at room temperature for 3 minutes. The cells left in the tube were cultured in a 5% CO_2_ incubator with RPMI 1640 medium supplemented with 10% fetal bovine serum (FBS). All experiments in the article used the CTCs of passage 5.

### Immunofluorescence

The cells were fixed in 4% (m:v) paraformaldehyde for 15 min at 4°C, washed with PBS, and then permeabilized with 0.3% Triton X-100 for 10 min at room temperature (RT). After non-specific binding was blocked with 1% BSA, the cells were incubated with primary antibody at 4°C in a humid chamber overnight. The cells were then incubated with a secondary antibody conjugated to FITC or TRITC for 1h at RT. After the cells were washed with PBS, images were taken with an Olympus inverted fluorescent microscope with CCD camera.

### Cell growth analysis

The cells were plated at 1×10^4^ cells per well in a 6-well plate and counted in triplicate using a blood counting chamber to generate a growth curve.

### Wound healing assay

We consulted the method in the report of Shi et al [71]. In short, a straight line scratch wound was created in a confluent monolayer of cells using a sterile 1ml serological pipette. And then washed and continue to culture the cells with RPMI 1640 medium. Images were photographed 100× magnification using an Olympus Imaging System Microscope at 0 h and 24 h. The scratch wound assay was performed in quadruplicate.

### Migration and invasion (Transwell) assays

The methods consulted the method in the report of Shi et al [71], briefly, 1×10^4^ cells were plated in the matrigel-coated upper chamber of a Corning Costar chamber (Corning, USA). The reservoir of the chamber was filled with complete medium and the cells were cultured continually. 16 hours later, the cells that remained in the top membrane surface were wiped out. The cells that had migrated to the lower surface were stained with crystal violet. Images of migratory cells were photographed 100× magnification using an Olympus Imaging System Microscope. The cell migration assays were performed in triplicate

### Pulmonary metastasis formation by intravenous injection

The mice were injected intravenously with 2×10^5^ cells. Two weeks later, the mice were euthanized and then dissected. The numbers of metastatic tumors contained in their lungs were counted.

### Immunohistochemical staining

All normal or prostate cancer samples from patients were obtained from the Department of Urology and diagnosed by the Department of Pathology of Xijing Hospital. The expression of TOPK was detected in these samples by immunohistochemistry. The tissue sections were de-paraffinized and rehydrated in PBS. The sections were subjected to antigen retrieval in 10 mM sodium citrate buffer, pH 9.0 for 20 minutes at 100°C and washed in PBS. Endogenous peroxidases were quenched in PBS containing 1.5% H_2_0_2_ for 30 minutes. The samples were rinsed in PBS, blocked in PBS containing 5% goat serum, and incubated with antibodies (1:200) to TOPK at RT for 2 hours. The samples were washed in PBS and incubated with a secondary goat anti-mouse antibody (1:500) for 1 hour and incubated with 3, 3′-Diaminobenzidine (DAB) substrate within 3 minutes.

After the mice were euthanized, their lungs and tumors were fixed in 4% formalin, routinely processed, and embedded in paraffin. Sections of 5-μm were placed on glass slides for hematoxylin & eosin staining and immunohistochemistry (IHC). Microwave heat-induced epitope retrieval in citrate buffer was used for IHC. The tumor and lung sections were stained with the TOPK antibody. Images were obtained at 40× and 400× magnification using an Olympus Imaging System Microscope.

### Western blotting

Cells (7×10^5^) were cultured in 10-cm dishes to 70%–80% confluence and harvested in 300 μl RIPA buffer. The samples were sonicated 3 times for 15 seconds and centrifuged at 13,000 rpm for 15 minutes. The quantity of protein was determined by the Bradford method. The samples (30-50 μg protein) with 5×SDS loading buffer were heated at 95°C for 10 minutes, cooled on ice, and then were loaded into each lane of a 10% SDS polyacrylamide gel for electrophoresis (SDS-PAGE) and subsequently transferred onto a PVDF transfer membrane (Millipore, Billerica, MA, USA). Antibody-bound proteins were detected by chemiluminescence (BIO-RAD, USA).

### Lentiviral infection

The lentiviral expression vectors including CTC-shTOPK or CTC-shMOCK and packaging vectors including pMD2.0G and psPAX were purchased from Addgene, Inc. (Cambridge, MA, USA). To prepare TOPK viral particles, viral vectors and packaging vectors (pMD2.0G and psPAX) were transfected into HEK293T cells using Lipofectamine 2000 following the manufacturer's suggested protocol. The transfection medium was changed 4 hours after transfection, and then the cells were cultured for 36 hours. The viral particles were harvested by filtration using a 0.22-μm syringe filter, then combined with 8 mg/mL polybrene, and used to infect 60% confluent CTCs overnight. The cell culture medium was replaced with fresh complete growth medium after 24 hours, and the cells were selected with puromycin (1.5 mg/mL) after 36 hours. The selected cells were used for experiments.

### Antibodies and reagents

PBK/TOPK mouse monoclonal antibody was purchased from Santa Cruz Biotechnology (Santa Cruz, CA, USA), phospho-p44/42 MAPK (Erk1/2) rabbit monoclonal antibody, phospho-AKT (Ser473) rabbit monoclonal antibody, phospho-PTEN (ser380/Thr382/382) mouse monoclonal antibody, and EpCAM (VU1D9) mouse monoclonal antibody conjugated to Alexa Fluor 594 were purchased from Cell Signaling Technology, Inc. (Beverly, MA, USA). Cytokeratin 19 rabbit monoclonal antibody was purchased from Abcam, Inc. (Cambridge, MA, USA), CD45 rabbit monoclonal antibody was purchased from Biosen, Inc. (Beijing, China), goat anti-mouse IgG conjugated to FITC and TRITC were purchased from Biosen, Inc. (Beijing, China), goat anti-rabbit IgG conjugated to FITC and TRITC were purchased from Biosen Inc. (Beijing, China), HRP-labeled goat anti-mouse IgG(H+L) and goat anti-rabbit IgG(H+L) were purchased from SinoBio, Inc. (SuZhou, China), and 4,6-diamino-2-phenyl indole (DAPI) was purchased from Beyotime, Inc. (Beijing, China), Diaminobenzidine (DAB) substrate was purchased from Dako Denmark A/S (Glostrup, Denmark), packaging vectors including pMD2.0G and psPAX were purchased from Addgene Inc. (Cambridge, MA, USA), polybrene were purchased from EMD Millipore (Billerica, MA, USA), puromycin were purchased from Sigma-Aldrich (St Louis, MO, USA).

### Statistical analysis

Significant differences were determined by one-way analysis of variance (ANOVA).

Correlations were determined with Pearson's x^2^ test using SAS9.2 software. All statistical tests were two-sided, and P < 0.05 was considered significant (*P < 0.05, **P < 0.01, ***P < 0.001).
